# Pseudohypoxia-Stabilized HIF2α Transcriptionally Inhibits *MNRR1*, a Druggable Target in MELAS

**DOI:** 10.3390/cells14141078

**Published:** 2025-07-15

**Authors:** Neeraja Purandare, Vignesh Pasupathi, Yue Xi, Vikram Rajan, Caleb Vegh, Steven Firestine, Tamas Kozicz, Andrew M. Fribley, Lawrence I. Grossman, Siddhesh Aras

**Affiliations:** 1 Center for Molecular Medicine and Genetics, School of Medicine, Wayne State University, Detroit, MI 48201, USA; 2 Department of Pediatrics, School of Medicine, Wayne State University, Detroit, MI 48201, USA; 3 Department of Pharmaceutical Sciences, Eugene Applebaum College of Pharmacy and Health Sciences, Wayne State University, Detroit, MI 48201, USA; 4 Department of Genetics and Genomic Sciences, Icahn School of Medicine at Mount Sinai, New York, NY 10029, USA; 5 Karmanos Cancer Institute, Wayne State University, Detroit, MI 48201, USA; 6 Department of Obstetrics and Gynecology, School of Medicine, Wayne State University, Hutzel Women’s Hospital 3990 John R. Street, 7 Brush North, Detroit, MI 48201, USA

**Keywords:** drug repurposing, heteroplasmy, hypoxia

## Abstract

The mitochondrial regulator MNRR1 is reduced in several pathologies, including the mitochondrial heteroplasmic disease MELAS, and genetic restoration of its level normalizes the pathological phenotype. Here, we investigate the upstream mechanism that reduces MNRR1 levels. We have identified the hypoxic regulator HIF2α to bind the MNRR1 promoter and inhibit transcription by competing with RBPJκ. In MELAS cells, there is a pseudohypoxic state that transcriptionally induces *HIF2α* and stabilizes HIF2α protein. MELAS cybrids harboring the m.3243A > G mutation display reduced levels of prolyl hydroxylase 3 (PHD3), which contributes to the HIF2α stabilization. These results prompted a search for compounds that could increase MNRR1 levels pharmacologically. The screening of a 2400-compound library uncovered the antifungal drug nitazoxanide and its metabolite tizoxanide as enhancers of *MNRR1* transcription. We show that treating MELAS cybrids with tizoxanide restores cellular respiration, enhances mitophagy, and, importantly, shifts heteroplasmy toward wild-type mtDNA. Furthermore, in fibroblasts from MELAS patients, the compound improves mitochondrial biogenesis, enhances autophagy, and protects from LPS-induced inflammation. Mechanistically, nitazoxanide reduces HIF2α levels by increasing PHD3. Chemical activation of MNRR1 is thus a potential strategy to improve mitochondrial deficits seen in MELAS. Finally, our data suggests a broader physiological pathway wherein two proteins, induced under severe (1% O2; HIF2α) and moderate (4% O2; MNRR1) hypoxic conditions, regulate each other inversely.

## 1. Introduction

Mitochondria are well known to be genetic hybrids, comprising products of nuclear DNA (nDNA) and mitochondrial DNA (mtDNA) [1,2]. Mutations in mtDNA, and its multicopy nature in which thousands of copies can be present in a diploid cell, give rise to a mixture of wild-type and mutant copies, termed heteroplasmy. Although there are diseases of homoplasmy, where only mutant mtDNA is present (e.g., LHON [3]), many mtDNA diseases are heteroplasmic like MELAS (Mitochondrial Encephalomyopathy, Lactic Acidosis and Stroke-like episodes), a rare genetic disease that affects multiple organs. In such cases of a pathogenic mtDNA mutation, the higher the heteroplasmy level, the more severe the phenotype [4,5,6,7]. As a result, reducing the heteroplasmy level represents a viable treatment strategy. Doing so can involve increasing the amount of wild-type mtDNA, reducing the number of mutant copies, or both.

Several pathogenic mtDNA mutations are specifically associated with the MELAS syndrome, the most common being m.3243A > G in the mitochondrial *MT-TL1* gene that codes for tRNA^Leu(UUR)^. The mutation causes hypophysiological mitochondrial protein translation and synthesis and can attain heteroplasmy levels >50% [8]. Mitochondria that cannot adequately translate and assemble electron transport chain (ETS) subunits produce insufficient energy to meet the requirements of their resident tissues along with other deficiencies, resulting in the characteristic MELAS multi-organ dysfunction whose sequelae are difficult to treat [9]. For example, the overall energy deficiency stimulates mitochondrial proliferation in endothelial cells, precipitating angiopathy and impaired blood perfusion that exacerbate organ damage and the stroke-like episodes [10]. Deficiency of nitric oxide (NO), which regulates smooth muscle relaxation, contributes to these sequelae, including hypertension, fatigue, and memory loss. Treatments that increase NO levels are common clinical approaches currently under study [11]. However, there are no specific standard treatments available to MELAS patients and many die between the ages of 10 and 35, underscoring a considerable unmet patient need [12].

Mitochondrial Nuclear Retrograde Regulator 1 (MNRR1; also called CHCHD2, PARK22, AAG10) is a biorganellar regulator of mitochondrial and nuclear function. MNRR1 was discovered in a computational screen to identify factors regulating the ~90 proteins that comprise the oxidative phosphorylation complex [13]. Work since then has revealed that mitochondrial MNRR1 binds to cytochrome *c* oxidase (COX) to activate respiration [14,15] and interacts with Bcl-xL to impede the extrinsic apoptosis cascade [16]. Nuclear located MNRR1, along with the protein RBPJκ, functions to activate transcription by binding to the conserved oxygen-responsive element (ORE) of its own promoter as well as to a host of other stress-response genes [17,18]. Recent work from our group demonstrated that ectopic expression of MNRR1 could rescue the MELAS mitochondrial phenotype in vitro by increasing oxidative phosphorylation (OXPHOS) and the expression of CREBH target genes, which led to a significant decrease in heteroplasmy [18]. We also found that the transcription of MNRR1 was inhibited in MELAS cybrid cells. Thus, we sought small molecules that could activate transcription of *MNRR1* (and its target genes) to improve MELAS heteroplasmy and its associated OXPHOS pathologies. We identified nitazoxanide as an activator of MNRR1 transcription and uncovered a novel mechanism by which MNRR1 transcription is inhibited in MELAS, and which is averted by the drug to enhance transcription.

## 2. Methods

### 2.1. Cell Lines

The human embryonic kidney cell line HEK293, the triple-negative breast cancer cell line MDA-MB-468, the human first trimester placental cells HTR8/SVNeo (HTR), SHSY5Y cells, and HMC3 were obtained from the ATCC (Manassas, VA, USA). The MELAS cells (143B human osteosarcoma cybrid) were a kind gift from Dr. Douglas Wallace. The HEK293 and human fibroblast cells were cultured in DMEM with L-glutamine and D-Glucose (Gibco, Billings, MO, USA) supplemented with penicillin-streptomycin (HyClone, Logan, UT, USA) and 10% fetal bovine serum (FBS) (Sigma-Aldrich, St. Louis, MO, USA); the MDA-MB-468 cells were cultured as above, with the addition of 1 mM pyruvate; the HTR cells were cultured in Roswell Park Memorial Institute Medium (RPMI) (HyClone, Logan, UT, USA), supplemented with 5% FBS plus penicillin-streptomycin; and the MELAS cells were grown in DMEM with 1 mM pyruvate supplemented with non-essential amino acids (Gibco, Billings, MO, USA), 50 μg/mL uridine, and 10% FBS. The SHSY5Y cells were cultured DMEM/F12 with L-glutamine and D-Glucose (Corning, Corning, NY, USA), supplemented with Penicillin-Streptomycin (HyClone, Logan, UT, USA) and 10% FBS.

### 2.2. Chemicals

Tizoxanide, nitazoxanide, and genetisate were obtained from Selleckchem (Houston, TX, USA), Phenyl-4-aminosalicylic acid was obtained from A2Bchem (San Diego, CA, USA), 2-Amino 5-nitrothiazole was from Sigma Aldrich (Burlington, MA, USA), and the remaining compounds (4-Aminobenzanilide, 4-Amino salicylic acid, benzanilide, phenyl benzoate, and phenyl salicylic acid) were from Santa Cruz Biotechnology (Dallas, TX, USA). All compounds were solubilized in DMSO (used as vehicle control in all experiments with these compounds). LPS (Lipopolysaccharide from *Escherichia coli* 0111:B4) was purchased from Invivogen (San Diego, CA, USA).

### 2.3. Plasmids

The *MNRR1* promoter luciferase reporter plasmid and pRL-SV40 *Renilla* luciferase expression plasmids have been described previously [14]. The HRE-luciferase was a gift from Navdeep Chandel (Addgene, Watertown, MA, USA, plasmid # 26731 http://n2t.net/addgene:26731 accessed on 21 March 2025; RRID:Addgene_26731), [19]). The HIF2alpha-pcDNA3 (plasmid # 18950 [20], http://n2t.net/addgene:18950 accessed on 21 March 2025; RRID:Addgene_18950), PHD1-pcDNA3 (plasmid # 18961, [21]; http://n2t.net/addgene:18961 accessed on 21 March 2025; RRID:Addgene_18961), and PHD3-pcDNA3 (plasmid # 18960 [21]; http://n2t.net/addgene:18960 accessed on 21 March 2025; RRID:Addgene_18960) plasmids were a gift from William Kaelin (Addgene, Watertown, MA, USA). The constitutively active version of RBPJκ has been previously described [17]. All expression plasmids were purified using the EndoFree plasmid purification kit from Qiagen (Germantown, MD, USA).

### 2.4. Stable Cell Line Generation

HEK293 or MDA-MB-468 cells were transfected with *MNRR1*-promoter luciferase, selected with 0.5 μg/mL (for 293) or 1 μg/mL (for 468) puromycin for 2 weeks, and subcloned by limiting dilution. MDA-MB-468-MNRR1-luc and HEK293-MNRR1-luc cells from multiple clones (5000–7500 cells) were plated in 100 μL of complete medium overnight and treated with 10 μM of each compound from the library for 24 h. The medium was aspirated from each well to a volume of 25 μL, and 25 μL Bright Glo (Promega, Madison, WI, USA) luciferase detection reagent was added to each well 10 min prior to luminescent determination with a FlexStation 3 Multimode Microplate Reader (Molecular Devices, San Jose, CA, USA). In the absence of a known MNRR1-inducing small molecule, we chose MNRR1 overexpression since MNRR1 induces its own expression [14], as a potential positive control. The Z’ factor is a value used to identify strong candidates and can be calculated using a previously described method [22]. The range of this value is negative infinity to 1, with >0.5 as a very good assay, >0 an acceptable assay, and <0 an unacceptable assay. The values obtained for this screen were >0, but we were unable to identify any candidates with strong values of 0.5 to 1.

### 2.5. MicroSource Spectrum Collection

Despite the low Z’ values for MDA-MB-468-MNRR1-luc and HEK293-MNRR1-luc, pilot screens were performed with both using the MicroSource Spectrum Collection (Gaylordsville, CT, USA). The Spectrum Collection comprises ~2400 small molecules and natural products that are known drugs or otherwise biologically well-characterized. This library contains a manageable number of compounds in 10 mM DMSO stocks that can be tested without the need for advanced liquid handling. Additionally, the use of biologically well-characterized compounds facilitates the rapid identification of pathways and signaling networks likely to be of interest to the investigator. Dry powder stocks of the compounds that provided the most robust response in both cell lines and that did not have chemical liabilities that would preclude their use in cultured cells or, potentially, in human subjects, were then obtained from commercial sources. For HTS, cells were plated, treated, and measured for luciferase expression as described for clone identification; all compounds were added to a final concentration of 10 μM and MNRR1-luciferase expression was measured after 24 h.

Since highly expressing clones that provided a Z’ value between 0.5 and 1.0 were elusive and there is a general lack of MNRR1 activators that could be used as positive controls, the criterion of accepting modestly enhanced transcription (1.8–2.8-fold) in each of two cell lines was used. Thus, a pilot screen with the MicroSource Spectrum Collection was performed in MNRR1-luciferase clones from MDA-MB-468-*MNRR1*-luc and HEK-293-*MNRR1*-luc cells. Compounds enhancing transcription in both cell lines were considered for further scrutiny with orthogonal assays to evaluate *MNRR1* gene and protein expression. Nitazoxanide emerged as a validated MNRR1 activator and was studied further using in vitro assays in MELAS cell lines and primary fibroblasts to determine whether chemically induced MNRR1 expression could improve known human pathologic mitochondrial deficiencies. In addition to the lack of a known positive control, we hypothesize that the low Z’ values observed with our *MNRR1*-luciferase cell lines were due to the relatively low levels of MNRR1 expressed at baseline in monolayer cultures of MDA-MB-468 and HEK293.

### 2.6. Transient Transfection of MELAS Cells

MELAS cells were transfected with the indicated plasmids using TransFast transfection reagent (Promega, Madison, WI, USA) according to the manufacturer’s protocol. A TransFast–DNA ratio of 3:1 in complete medium was used. Following incubation at room temperature for ~15 min, the cells were overlaid with the mixture. The plates were incubated for overnight at 37 °C followed by replacement with fresh complete medium and further incubation for the indicated time.

### 2.7. Real-Time Polymerase Chain Reaction (RT-PCR)

Total cellular RNA was extracted from MELAS cells with a RNeasy Plus Mini Kit (Qiagen, Germantown, MD, USA) according to the manufacturer’s instructions. Complementary DNA (cDNA) was generated by reverse transcriptase polymerase chain reaction (PCR) using the ProtoScript^®^ II First Strand cDNA Synthesis Kit (New England Biolabs, Ipswich, MA, USA). Transcript levels were measured by real time PCR using SYBR green on an ABI 7500 system. Real-time analysis was performed by the ∆∆^Ct^ method [23]. The primers used were *MNRR1* forward: 5′-CACACATGGGTCACGCCATTACT-3′, reverse: 5′-TTCTGGGCACACTCCAGAAACTGT-3′; 18s forward: 5′-CCAGTAAGTGCGGGTCATAA-3′, reverse: 5′-GGCCTCACTAAACCATCCAA-3′; *PHD1 (EGLN2*) forward: 5′-ACATCGAGCCACTCTTTGAC-3′, reverse: 3′-TCCTTGGCATCAAAATACC-5′ [24]; *PHD3* (*EGLN3*) forward: 5′-TCAAGGAGAGGTCTAAGGCAA-3′, reverse: 3′-ATGCAGGTGATGCAGCGA-5′ [25]; and *HIF2α* (*EPAS1*) forward: 5′-CACCAAGGGTCAGGTAGTAA-3′, reverse: 3′-AACACCACGTCATTCTTCTC-5′.

### 2.8. Luciferase Reporter Assay

Luciferase assays were performed with the dual-luciferase reporter assay kit (Promega, Madison, WI, USA). Briefly, cells were lysed in 1X passive lysis buffer (Promega, Madison, WI, USA) and 25 μL of lysate was used for assay with a tube luminometer using an integration time of 10 s. Transfection efficiency was normalized with the co-transfected pRL-SV40 *Renilla* luciferase expression plasmid [15,17].

### 2.9. Immunoblotting

Immunoblotting was performed as described previously [15,17]. Cell lysates for immunoblotting were prepared using RIPA buffer (Abcam, Waltham, MA, USA) and included a protease and phosphatase inhibitor cocktail (Sigma-Aldrich, St. Louis, MO, USA). Total protein extracts were obtained by centrifugation at 21,000× *g* for 30 min at 4 °C. The clear supernatants were transferred to new tubes and quantified using the Bradford reagent with BSA as standard (BioRad, Hercules, CA, USA). Equal amounts of cell lysates were separated by sodium dodecyl sulfate–polyacrylamide gel electrophoresis (SDS–PAGE), transferred to PVDF membranes (BioRad), and blocked with 5% non-fat dry milk. Incubation with primary antibodies (used at a concentration of 1:500) was performed overnight at 4 °C. The PGC1α (Catalog # 2178), PINK1 (6946), LC3A/B (12741), phosphoserine-65 ubiquitin (62802), HIF2α (59973), TOM20 (72610), GAPDH (8884), actin (12748), and tubulin (9099) antibodies were obtained from Cell Signaling (Danvers, MA, USA).

The MNRR1 (19424-1), MTCO2 (55070-1), PHD1 (12984-1), and PHD3 (18325-1) antibodies were obtained from Proteintech (Chicago, IL). Incubation with secondary antibodies (1:5000) was performed for 2 h at room temperature. For detection after immunoblotting, the SuperSignal™ West Pico PLUS substrate or SuperSignal™ West Femto Maximum Sensitivity Substrate (ThermoFisher, Waltham, MA, USA) was used to generate chemiluminescence signal, which was detected with X-Ray film (RadTech, Vassar, MI, USA).

### 2.10. Immunofluorescence

Cells plated on glass cover slips were fixed with 3.7% formaldehyde (prepared in 1X PBS) at room temperature for 15 min, followed by permeabilization with 0.15% Triton X-100 (prepared in distilled water) for 2 min, and then blocked with 5% bovine serum albumin (BSA) (prepared in 1X PBS, 0.1% TWEEN-20 (PBST)) for 1 h at room temperature. Cells were washed with PBST then incubated for 1 h at room temperature in primary antibody solution containing Coralite^®^ 594 conjugated mouse monoclonal anti-CHCHD2 IgG (1:100, Proteintech, Cat. No. CL594-66302), prepared in PBST. Cells were washed 3 times with PBST for 5 min each and mounted with Vectashield vibrance with DAPI (Cat. # H-1800-10, Vector Labs, Newark, CA, USA). Cells were imaged at 63× on the confocal 60 μm disk setting with the BioTek Cytation C10 using the, software (version 3.11.19, Agilent, Santa Clara, CA). Six fields for each group were taken and z-stacks of 25 slices (±3 slices) were performed for each field followed by a z-projection and image deconvolution. Corrected total fluorescence for each field to determine MNRR1 content was calculated using FIJI (Version 2.14.0/1.54f, National Institutes of Health).

### 2.11. Mitochondrial DNA Levels

Total genomic DNA was isolated from cells expressing each of the mutants using the Invitrogen PureLink Genomic DNA Mini Kit (Thermo Fisher Scientific, Waltham, MA, USA, Catalog # K1820-01) and analyzed by real-time PCR as above. The primer sequences used to amplify mtDNA and GAPDH were as follows: mtDNA forward: 5′-CCTCCCTGTACGAAAGGAC-3′; reverse: 5′-GCGATTAGAATGGGTACAATG-3′; GAPDH forward: 5′-GAGTCAACGGATTTGGTCGT-3′; reverse: 5′-TTGATTTTGGAGGGATCTCG -3′.

### 2.12. Restriction Enzyme Digestion

DNA was analyzed for the MELAS mutation as described previously [18]. The A → G mutation creates a new HaeIII site at position 3243 that can be amplified by PCR using primers corresponding to the light-strand positions 3116 to 3134 and to the heavy-strand positions 3353 to 3333. Equal amounts of the resulting products were digested with the restriction enzyme HaeIII (New England Biolabs, Ipswich, MA, USA) overnight at room temperature and electrophoresed on a 2.5% agarose gel.

### 2.13. ROS Measurements

Total cellular ROS measurements were performed with CM-H2DCFDA (Life Technologies, Carlsbad, CA, USA). Cells were distributed into 96-well plates at 2.5 × 10^4^ cells per well and incubated for 24 h or as described in specific experiments. Cells were then treated with 10 μM CM-H_2_DCFDA in serum- and antibiotic-free medium for 1 h. Cells were washed twice in phosphate-buffered saline and analyzed for fluorescence on a BioTek Synergy H1 Microplate Reader (Agilent, Santa Clara, CA, USA).

### 2.14. Intact Cellular Oxygen Consumption and Measurement of ATP

Cellular oxygen consumption was measured with a Seahorse XF^e^24 Bioanalyzer (Agilent, Santa Clara, CA, USA). Cells were plated at a concentration of 2 × 10^4^ (MELAS cybrids) or 3.5 × 10^4^ (MELAS patient fibroblasts) per well a day prior to treatment and basal oxygen consumption was measured 48 h after treatments, as described [15,23]. For ATP levels, Agilent Seahorse ATP Real-Time rate assay kit was used as per the manufacturer’s instructions.

### 2.15. Chromatin Immunoprecipitation-qPCR (ChIP-qPCR)

Chromatin immunoprecipitation was performed as per the manufacturer’s instructions using SimpleChIP^®^ Enzymatic Chromatin IP Kit (Cell Signaling, Danvers, MA, USA, Catalog # 9002). Briefly, 2 × 10^7^ cells were fixed with formaldehyde to crosslink and chromatin was digested into ~150–900 bp fragments using a combination of micrococcal nuclease and sonication. A total 2% of the sample was stored as input control. This digested chromatin was immunoprecipitated using a HIF2α antibody (Cell Signaling, Danvers, MA, USA, Catalog # 59973). Samples were eluted and the crosslinking was reversed. The eluted DNA and the input controls were purified and tested for relative amplification using qPCR analysis. The primers used were as follows: forward: 5′-ATCTTCCGGTCTCCTCAGAA-3′; reverse: 3′-AAACCCTGCGATGGTCTCA-5′.

### 2.16. Statistical Analysis

All statistical analyses were performed with the two-sided Wilcoxon rank sum test using MSTAT version 6.1.1 (N. Drinkwater, University of Wisconsin–Madison). * *p*< 0.05; ** *p* < 0.005.

## 3. Results

### 3.1. High Throughput Screen to Identify MNRR1 Activators

**Figure 1:**
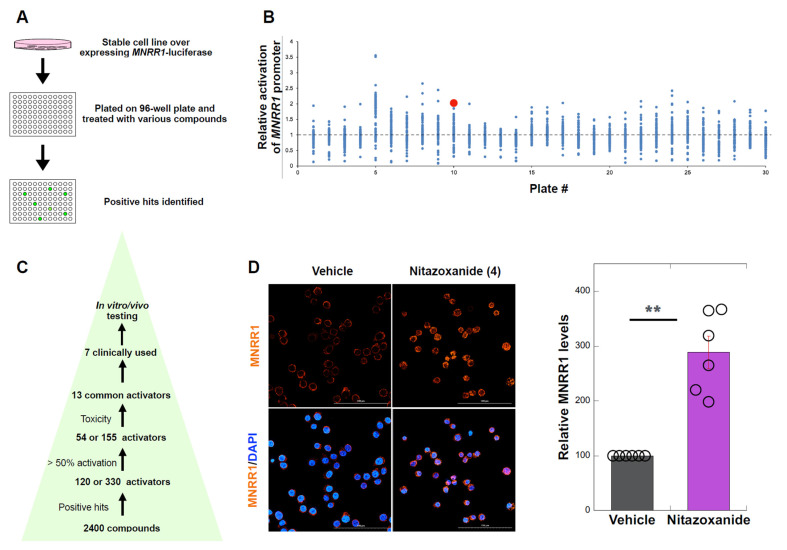
**High throughput screen to identify MNRR1 activators.** (**A**): Overview of the high-throughput screening process to identify activators of MNRR1. (**B**): Campaign view representing compounds that activate MNRR1. Control levels are represented as a dotted line. Each compound is shown as a blue dot and nitazoxanide is highlighted as a red dot. (**C**): Scheme for screening for strong activators of MNRR1 from high throughput screen. (**D**): MDA-MB-468 cells treated with Vehicle (DMSO) or nitazoxanide (10 μM) for 24 h were immunostained (MNRR1 = orange, DAPI = blue) and imaged at 63× using a confocal microscope. The scale bar represents 200 μm. The relative MNRR1 level is shown graphically. ** indicates *p* < 0.005.

We have previously shown that activation of MNRR1 using exogenous overexpression rescues mitochondrial deficit in MELAS cybrid cells and stable overexpression was able to shift heteroplasmy towards wild-type (WT) mtDNA [18]. Hence, we were interested in identifying the chemical activators of MNRR1 that could be repurposed as a therapeutic intervention in MELAS patients. To identify activators of MNRR1, we performed a screen of 2400 FDA-approved compounds using two independent cell lines, HEK293 and MDA-MB-468, stably expressing the MNRR1-promoter driven luciferase reporter (Figure 1A). We selected compounds that activated the reporter by at least 50% (Figure 1B), identifying 54 and 155 compounds on the HEK293 and MDA-MB-468 screens, respectively (Figure S1A). Thirteen compounds were common to both cell lines and, of these, 7 were selected based on their lower toxicity profile. Of these, six were selected based on clinical availability (Figure 1C). We then validated these compounds in a 143B osteosarcoma cell line (DW7) cybrid with ~73% MELAS mutant mtDNA (m.3243A > G) in which MNRR1 levels were reduced [18] (Figure S1B). We chose compound 4 since this was available as a clinical formulation that has been used for in vivo testing and shown to increase *MNRR1* transcripts and protein levels [26]. We also confirmed the increase in MNRR1 in one of the original cell lines used for identifying the activators—MDA-MB- 468 (Figure 1D) as well as in several other human cell lines (using tizoxanide, the active nitazoxanide metabolite—see below) (Figure S1C–E).

Nitazoxanide in cells is metabolized to tizoxanide (>97%) plus minor metabolites such as aminonitrothiazole and gentisate [27]. Only nitazoxanide- and tizoxanide-treated DW7 cells displayed a significant increase in the protein levels of MNRR1 (Figure S2A). To confirm that the effects were at the transcription level, we measured MNRR1 transcripts and observed that both compounds induced its transcription (Figure S2B).

### 3.2. MNRR1 Activation Using Tizoxanide Enhances Mitochondrial Biogenesis and Mitophagy to Shift Heteroplasmy in MELAS Cybrid Cells

**Figure 2:**
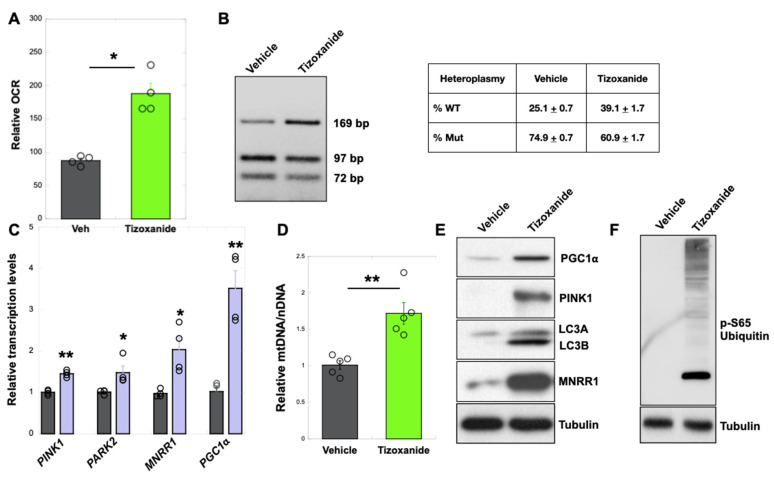
**MNRR1 activation by tizoxanide enhances mtDNA biogenesis and mitophagy to shift heteroplasmy in MELAS cells**. (**A**): Oxygen consumption measured from MELAS cybrid cells treated with vehicle (DMSO) or tizoxanide (10 μM) for 48 h (n = 4 biological replicates, error bars represent SE). (**B**): HaeIII restriction enzyme digestion of a PCR-amplified fragment of mtDNA harboring the m.3243A > G mutation from DW7 cells treated with vehicle (DMSO) or tizoxanide (10 μM) for 144 h). The table shows quantitation for n = 4 biological replicates, values depict mean ± SD. (**C**): Genes identified using qPCR of MELAS cybrid cells stably overexpressing EV or MNRR1. (**D**): MtDNA levels are shown relative to nuclear DNA (nDNA) (GAPDH) (n = 5 biological replicates, error bars represent SE). (**E**): Equal amounts of MELAS cybrid cell lysates, treated with vehicle (DMSO) or tizoxanide (10 μM) for 48 h, were separated on an SDS-PAGE gel and probed for the proteins shown. (**F**): Equal numbers of MELAS cells were treated as in (**E**), separated on an SDS-PAGE gel, and probed for phospho-ubiquitin (Ser65) levels. Tubulin was probed as a loading control. * indicates *p* < 0.05, ** indicates *p* < 0.005.

We first confirmed the effects of MNRR1 activation by measuring oxygen consumption, which was increased (Figure 2A). We had previously shown that MNRR1 overexpression induces homeostatic pathways such as mitophagy and mitochondrial biogenesis to aid in rescuing the phenotype. We had also shown that MNRR1 overexpressing cells display a reduction in heteroplasmy, making it an attractive therapeutic target for MELAS [18]. We therefore tested tizoxanide on MELAS cybrid cells and found it increased the proportion of WT mtDNA, by about 14% here as shown by HaeIII digestion of the mtDNA fragment harboring the MELAS point mutation (Figure 2B). Stable overexpression of MNRR1 in MELAS cybrid cells activated multiple homeostatic genes (Figure 2C) and restored healthy mitochondria, presumably in part by stimulating mtDNA synthesis (Figure 2D) by increasing PGC1α (Figure 2C,E) and enhancing mitophagy. Increased mitophagy is shown by an increase in PINK1 (Figure 2C,E), autophagosomal proteins LC3A and LC3B (Figure 2E), and by increased levels of the mitophagy marker pSer65 ubiquitin (Figure 2F) [28]. Taken together, these results suggest that tizoxanide increases MNRR1 levels and thereby induces the downstream pathways that rescue defective mitochondrial function in MELAS cybrid cells.

### 3.3. MNRR1 Activation Using Tizoxanide in MELAS Patient Fibroblasts Enhances Mitochondrial Function and Mitophagy and Protects from LPS-Induced Inflammation

**Figure 3:**
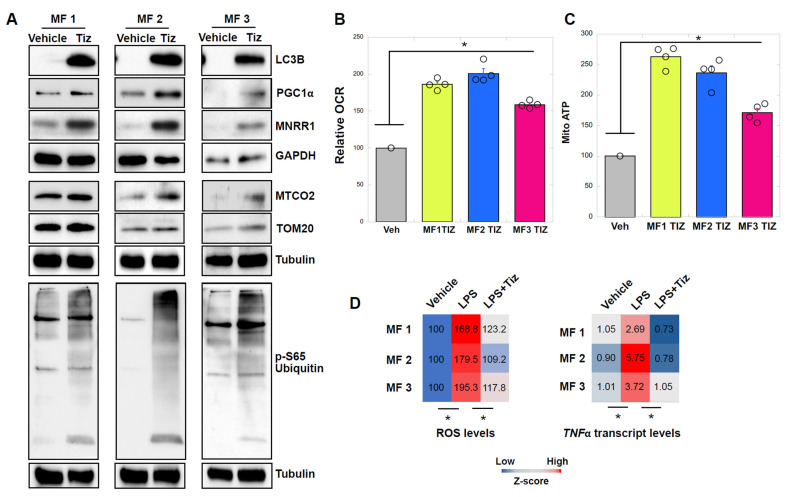
**MNRR1 activation using tizoxanide enhances mitochondrial function, mitophagy, and protects from LPS-induced inflammation in MELAS patient fibroblasts** (**A**): Equal amounts of lysates from MELAS patient fibroblast (MF 1, 2, or 3) lysates treated with vehicle (DMSO) or tizoxanide (10 μM) for 48 h were separated on an SDS-PAGE gel and probed for LC3B, PGC1α, MNRR1, MTCO2, TOM20, and phospho-ubiquitin (S65) levels. GAPDH or Tubulin was probed as a loading control. (**B**): Oxygen consumption measured from MELAS patient fibroblasts treated with vehicle (DMSO) or tizoxanide (10 μM) for 48 h (n = 4 biological replicates, mean ± SE). (**C**): Mitochondrial ATP rate measured in MELAS patient fibroblasts treated with vehicle (DMSO) or tizoxanide (10 μM) for 48 h (n = 4 biological replicates, mean ± SE). (**D**): Heatmap representation of ROS and TNFα transcript levels from MELAS patient fibroblasts treated with vehicle (DMSO), LPS (DMSO + 500 ng/mL LPS), or LPS plus tizoxanide (10 μM tizoxanide + 500 ng/mL LPS) for 24 h (n = 4 biological replicates). Values for each sample are shown. * indicates *p* < 0.05.

In primary fibroblasts from three independent MELAS patients, we found that activation of MNRR1 enhances mitophagy (as seen via LCB and pSer65 ubiquitin levels) and mitochondrial biogenesis (PGC1α, MTCO2, TOM20 levels) (Figure 3A). Furthermore, we found that OCR (Figure 3B) and mitochondrial ATP (Figure 3C) levels were enhanced. Since we recently found in a cell culture model that activation of MNRR1 resolved the effect of LPS-induced inflammation [29], and that mitochondrial diseases are associated with a pro-inflammatory phenotype [30,31,32], we also asked if we could rescue the effects of LPS in these patient fibroblasts. We found that LPS induces a pro-inflammatory response as judged by increased ROS and TNF levels, and that this response can be blocked by tizoxanide (Figure 3D), suggesting that MNRR1 activation is protective in these cells.

### 3.4. Nitazoxanide Acts by Reducing HIF2α Protein Levels in MELAS Cybrid Cells

**Figure 4:**
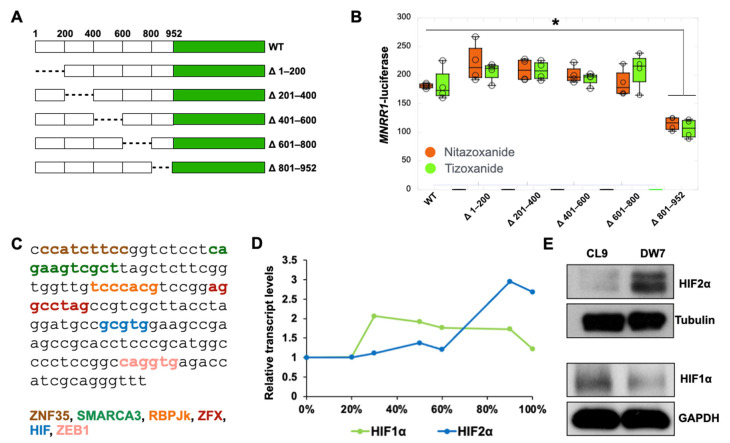
**Nitazoxanide acts by reducing HIF2α levels in MELAS cells.** (**A**): Schematic representation of deleted regions of the MNRR1 promoter. (**B**): Dual luciferase reporter assay using the MNRR1 promoter deletions in cells treated with vehicle (DMSO), nitazoxanide, or tizoxanide (10 μM) for 24 h (n = 4 biological replicates). (**C**): DNA sequence of region of 800–952 bp in the MNRR1 promoter, highlighting binding sites of the transcription factors shown. (**D**): Transcript levels of *HIF2α* and *HIF1α* in MELAS cybrid cells harboring different levels of heteroplasmy (0% to 100%). Data from [8]. (**E**): Protein levels of HIF2α and HIF1α in cybrid cells with 0% (CL9) and 73% MELAS heteroplasmy (DW7). * indicates *p* < 0.05.

**Figure 5:**
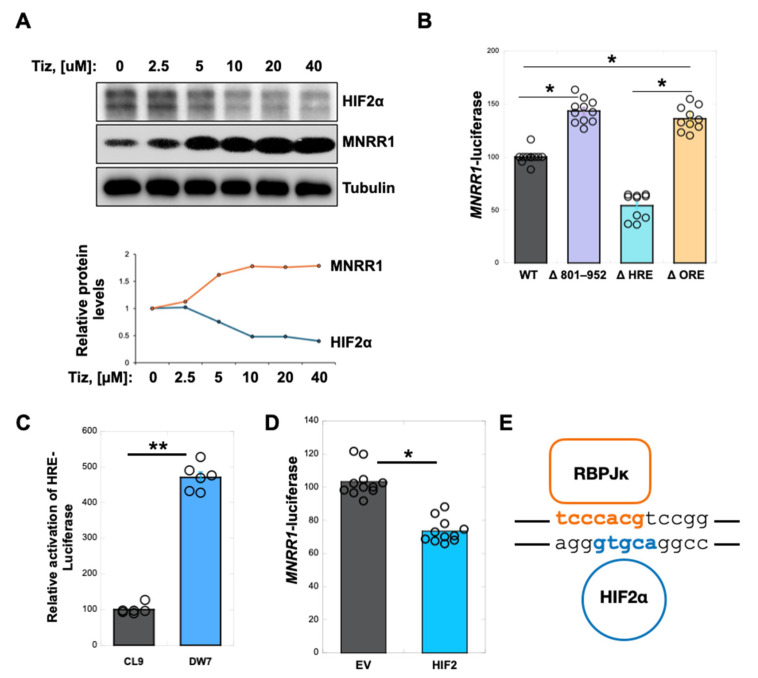
**The reduction in MNRR1 in MELAS cells is via HIF2α acting at the ORE and not the HRE.** (**A**): *Above*, equal amounts of MELAS cell lysates, treated with vehicle (DMSO) or increasing amounts of tizoxanide (2.5 to 40 μM) for 24 h, were separated on an SDS-PAGE gel and probed for HIF2α, MNRR1, and Tubulin. *Below,* protein quantification for MNRR1 and HIF2α relative to Tubulin. (**B**): Dual luciferase reporter assay showing relative activation of *MNRR1*-luciferase in MELAS cybrid cells overexpressing WT (Figure 4A), Δ 801–952 (Figure 4A), ΔORE (sequence in orange in Figure 4C), or ΔHRE (sequence in red in Figure 4C). (**C**): Dual luciferase reporter assay showing relative activation of *HRE*-luciferase levels in cybrid cells with 0% (CL9) and 73% MELAS heteroplasmy (DW7). (**D**): Dual luciferase reporter assay showing relative activation of *MNRR1*- luciferase levels in MELAS cybrid cells with 0% heteroplasmy (CL9) overexpressing EV (empty vector) or HIF2α. (**E**): Sequences in *MNRR1* promoter highlighting the binding sites for RBPJκ (orange) and HIF2α (blue) on opposite strands of DNA. * indicates *p* < 0.05, ** indicates *p* < 0.005.

Nitazoxanide was identified by screening a library for transcriptional activators of *MNRR1* using 952 bp promoter luciferase-expressing stable cell lines. We therefore sought to identify the region on the MNRR1 promoter that responds to nitazoxanide with MNRR1 induction. To this end, we generated 200 bp deletions in the *MNRR1* promoter and cloned them into the pGL4-basic luciferase vector (Figure 4A). Upon testing the responsiveness of each of these constructs to nitazoxanide and tizoxanide in MELAS cybrid cells, we observed that a deletion of the 801–952 region on the promoter (Δ801–952) failed to display activation (Figure 4B), suggesting that this promoter region was affected by nitazoxanide. Bioinformatic analysis of this region identified six bonafide binding sites for transcription factors (TFs)—Zeb1, HIF, ZFX, SMARCA3, ZNF35, and RBPJκ (Figure 4C). RBPJκ binds to the core 13 bp element that we previously characterized to be responsive to moderate hypoxia and labeled as the oxygen responsive element (ORE) [17]. Of the six [8], who initially characterized the MELAS cybrids, identified only HIF2α to be transcriptionally induced (Figure 4D). We assessed the level of both and found that HIF1α was not increased in the MELAS cells whereas HIF2α levels were higher in heteroplasmic MELAS cybrid cells (DW7) (Figure 4E) as compared to the control cybrids (CL9) [8,33]. To test whether tizoxanide was acting through HIF2α, we measured its protein levels and found that HIF2α was inversely proportional in a concentration-dependent manner to MNRR1 in MELAS cybrid cells treated with tizoxanide (Figure 5A). To assess whether HIF2α is acting specifically via the hypoxia response element (HRE), we generated a deletion of the HRE in the MNRR1 promoter (Figure 4C, blue). To our surprise, we found that deletion of the HRE could not reverse the inhibition in MELAS cells, whereas deletion of the ORE could rescue the effects (Figure 5B). To evaluate this confounding effect, we tested an HRE-harboring reporter in the MELAS cybrid cells (DW7) and found it to be more active than in the control cybrid cells (CL9) (Figure 5C). In the same control cybrid cells, we could also overexpress *HIF2α* and repress *MNRR1* transcription (Figure 5D). However, since this effect was not through the HRE in the *MNRR1* promoter (Figure 5B), we again examined the sequence of the 800–952 region on the promoter and now uncovered a second HRE in the reverse orientation on the opposite strand of the ORE where RBPJκ binds (Figure 5E), thus providing a possible explanation for the effects seen in Figure 5B. We previously showed that MNRR1 forms a required transcriptional complex with RBPJκ at the ORE and that constitutively active RBPJκ can bypass the need for MNRR1 to activate transcription [17].

### 3.5. RBPJk and HIF2α Compete for Binding at the ORE in the MNRR1 Promoter to Regulate Transcription

**Figure 6:**
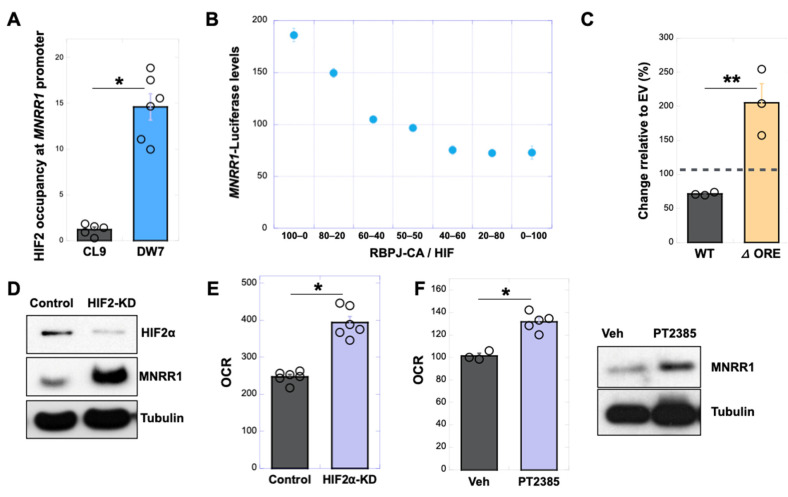
**RBPJκ and HIF2α compete for binding at the ORE in the MNRR1 promoter to regulate transcription.** (**A**): Chromatin immunoprecipitation-qPCR assessing binding of HIF2α to the endogenous *MNRR1* promoter in MELAS cybrid cells with 0% (CL9) and 73% heteroplasmy (DW7). (**B**): Dual luciferase reporter assay showing relative activation of *MNRR1*-luciferase levels in MELAS cybrid cells overexpressing varying proportions of constitutively active RBPJκ (RBP-CA) and HIF2α. (**C**): Dual luciferase reporter assay showing relative activation of *MNRR1*-luciferase levels in MELAS cybrid cells overexpressing the WT (Figure 4A) or ΔORE (sequence in orange in Figure 4C) promoter with EV (dotted line) or HIF2α. (**D**): Equal amounts of the control or HIF2α knockdown (KD) MELAS cybrid cell lysates were separated on an SDS-PAGE gel and probed for HIF2α and MNRR1 levels. Tubulin was probed as loading control. (**E**): Oxygen consumption measured from the control or HIF2α knockdown (KD) MELAS cybrid cells (n = 4 biological replicates, error bars represent SE). (**F**): *Left,* oxygen consumption measured from MELAS cybrid cells treated for 24 h with vehicle (DMSO) or PT2385 (10 μM), an inhibitor of HIF2α function. *Right*, equal amounts of MELAS cybrid cell lysates were treated with vehicle (DMSO) or PT2385 (10 μM) for 24 h, separated on an SDS-PAGE gel, and probed for MNRR1 levels. GAPDH was probed as a loading control. * indicates *p* < 0.05, ** indicates *p* < 0.005.

To dissect the effect of HIF2α and RBPJκ at the ORE, we first confirmed by chromatin immunoprecipitation that HIF2α can bind at the MNRR1 promoter (Figure 6A). Using a constitutively active version of RBPJκ (CA-RBPJκ), we could rescue defective transcription of MNRR1 in MELAS cybrid cells (Supplementary Figure S3A) and block these effects by overexpression of HIF2 (Supplementary Figure S3B). We also titrated RBPJκ and HIF2α in MELAS cybrid cells and found that HIF2α can compete with, and inhibit, transcription induced by RBPJκ (Figure 6B). Furthermore, a chemical inhibitor that blocks binding of RBPJκ to DNA (Auranonfin) was able to block the effects of MNRR1-induced transcription (Supplementary Figure S3C), whereas the effects of HIF2α overexpression were blocked by deletion of the ORE (Figure 6C). Taken together with a previously published report that HIF interacts with RBPJk to inhibit transcription [34], we hypothesize that nitazoxanide relieves the inhibitory effect of HIF2α at the ORE to facilitate transcriptional activation via RBPJκ. At the protein level, depletion of HIF2α in the MELAS cybrid cells increases MNRR1 (Figure 6D) and oxygen consumption (Figure 6E). As a further confirmation, we specifically inhibited HIF2α with the compound PT2385 [35] and observed a rebound increase in both OCR and MNRR1 protein in MELAS cybrid cells (Figure 6F). Consequently, we propose that HIF2α acts in two ways to regulate *MNRR1* transcription as follows: (i) it binds to the HIF site shown in blue (Figure 4C) in the 800–952 bp region of the *MNRR1* promoter to activate transcription, and (ii) it binds on the complementary stand of the ORE site shown in orange (Figure 5E) to inhibit transcription. A balance between these mechanisms is imposed by the ratio of RBPJκ to HIF, as is suggested in Figure 6B.

### 3.6. PHD3 Levels Are Reduced in MELAS Cybrid Cells and Enhanced by Nitazoxanide to Increase MNRR1 Levels

**Figure 7:**
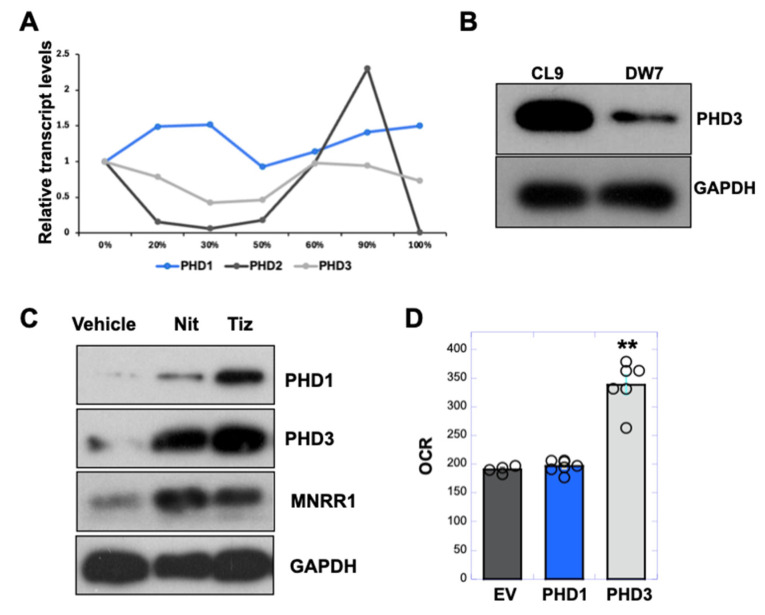
**PHD3 levels are reduced in MELAS cybrid cells and enhanced by nitazoxanide to increase MNRR1 levels.** (**A**): Transcript levels of *PHD1*, *2*, and *3* in MELAS cybrid cells harboring the levels of heteroplasmy shown (data from [8]). (**B**): Protein levels of PHD3 in MELAS cybrid cells with 0% (CL9) and 73% heteroplasmy (DW7). GAPDH was probed as loading control. (**C**): Equal amounts of MELAS cybrid cell lysates, treated with vehicle (DMSO), nitazoxanide, or tizoxanide (10 μM) for 24 h, were separated on an SDS-PAGE gel and probed for PHD1, PHD3, MNRR1, and GAPDH. (**D**): Oxygen consumption measured from MELAS cybrid cells overexpressing PHD1 or PHD3 (n = 4 biological replicates, error bars show SE). ** indicates *p* < 0.005.

The absence of any change in *HIF2α* transcript levels with nitazoxanide and tizoxanide (Supplementary Figure S4) suggested that HIF was showing greater protein stability. Hence, we assessed the levels of all three prolyl hydroxylases (*PHD1*, *2*, and *3*) [36,37] in the MELAS cybrid cells using published transcriptomics data (Figure 7A) [8]. Of these, we found that only PHD2, which shows specificity for HIF1α, was increased at high heteroplasmy, consistent with the reduction in HIF1α in DW7 versus CL9 cells (Figure 4E). Next, we assessed the levels of PHD3 and found that it was reduced in the DW7 MELAS cells (Figure 7B). Since HIF2α is increased in DW7 cells (Figure 4E), we examined the role of PHD1 and PHD3. We found that PHD3 is the most increased by tizoxanide at the protein level (Figure 7C) and, when overexpressed, uniquely stimulates respiration in DW7 cells (Figure 7D), suggesting that PHD3 may be the prolyl hydroxylase responsible for targeting HIF2α. PHD2 was not evaluated here, as studies have shown this enzyme to be selective for HIF1 [28,29], whose levels in MELAS cells were not correlated with heteroplasmy [8].

## 4. Discussion

We previously showed that MELAS cells contain a reduced amount of MNRR1 and that genetically restoring the amount alleviated much of the pathophysiological phenotype, such as reduced energy generation and increased ROS [18]. This suggested that activation of MNRR1 could improve mitochondrial deficits associated with MELAS and stimulated a search for a small molecule that would restore MNRR1 levels. We discovered the *MNRR1* transcriptional activator nitazoxanide by screening a drug and natural products chemical library. We found that nitazoxanide, along with its metabolic breakdown product tizoxanide, can restore expression and thereby function in MELAS cybrid cells and, importantly, in primary fibroblasts from MELAS patients. In seeking to identify the drug’s mechanism of action, we uncovered that MELAS cells contain increased amounts of HIF2α at normoxia, and that HIF2α binds at the *MNRR1* promoter to inhibit transcription. Surprisingly, the higher levels of HIF2α are a sum total of increased transcription [8] and enhanced stability imparted due to reduced levels of prolyl hydroxylase 3, the enzyme responsible for its degradation.

In parasites nitazoxanide inhibits pyruvate–ferredoxin oxidoreductase (PFOR), a key enzyme utilized by anaerobes in the oxidative decarboxylation of pyruvate to acetyl-CoA and CO2, whereas in humans, nitazoxanide has no known target. We first identified the region of the MNRR1 promoter where nitazoxanide acts and then narrowed to the specific transcription factors—HIF2α and RBPJκ—that bind to overlapping regions in the ORE part of the promoter to regulate transcription (Figure 5E). We have previously shown that RBPJκ binds to and mediates transcription at the ORE [17] and now identify a second factor—HIF2α—that can bind to the MNRR1 promoter (Figure 5E) to regulate transcription. These factors compete with each other; for example, active RBPJκ can enhance transcription of *MNRR1* and HIF2α competes with and represses this effect. In MELAS cybrid cells, which show a pseudohypoxic state, expressing higher levels of HIF2α, *MNRR1* is transcriptionally inhibited. With regard to the action of nitazoxanide, we propose a novel mechanism of post-translational regulation as follows: we found that PHD3, the prolyl hydroxylase that is largely responsible for targeting HIF2α for degradation by the ubiquitin proteasome [38,39], is upregulated by tizoxanide, thereby increasing MNRR1 levels. Since the anti-inflammatory effect of nitazoxanide was explicitly shown to depend on MNRR1 [26], this effect of nitazoxanide will be an important focus of future studies.

In non-heteroplasmic cells, overexpression of HIF2α inhibits the MNRR1 promoter, suggestive of a broader physiological regulation between players induced at severe and moderate hypoxia. HIF2α is the chronic responder at oxygen tensions <1% whereas MNRR1 responds to oxygen tensions of around 4%. This suggests a potential mechanism by which the drivers of hypoxia-responsive genes regulate each other to facilitate homeostasis. Furthermore, it is likely that the level of induction differs depending on the requirement of a particular gene during distinct oxygen tensions and depending on the driver.

We analyzed heteroplasmy at multiple time points and found that the optimum time point at which the shift was observed was 144 hr. The wild-type band (169 bp) intensity started increasing as early as 96 h and was visibly higher by 144 h (Figure 2B). The patient fibroblasts did not show a shift in heteroplasmy after tizoxanide treatment, unlike the shift toward wild-type seen in 73% MELAS cybrid cells (Figure 2B). This may result from the low heteroplasmy levels (<50%) in the patient fibroblasts, consistent with distinct nuclear responses for each heteroplasmy range, previously seen in the cybrid model of MELAS [8]. Lower heteroplasmy levels may not induce sufficient stress in these glucose-grown cells to alter heteroplasmy.

Nitazoxanide is the prodrug formulation of tizoxanide, a commonly used FDA-approved antiprotozoal effective against diarrheal symptoms caused by *Giardia* or *Cryptosporidium* [40,41]. Nitazoxanide is deacetylated in vivo to tizoxanide, which has antioxidant properties and is a known inhibitor of iNOS [42]. Both properties are likely major contributors for the observed improvements in lung and other organ damage in SARS-CoV-2 patients [43]. Consistently, MNRR1 depletion increases ROS production [14]. Thus, nitazoxanide has the potential to also alleviate MELAS beyond activation of *MNRR1* transcription. Furthermore, this recently rekindled interest in the broad spectrum of tizoxanide applications to human health has led to a new inquiry.

The generation of more plasma-stable congeners that replace the acetyl group substituent attached to the hydroxyl group with a more stable formamyl group [44], as well as novel approaches for tizoxanide quantification in vitro and in vivo, have recently been published [45,46]. Further studies will elucidate how increased plasma concentrations and systemic exposure to the active tizoxanide moiety will be tolerated and distributed in in vivo models of infection, cancer, and other maladies with unmet needs.

In summary, we have identified nitazoxanide and its metabolite tizoxanide as a drug that stimulates *MNRR1* transcription and shown that it reduces heteroplasmy in a cell culture model of MELAS and improves the phenotype in MELAS patient fibroblasts. Given its established safety profile, it could be usefully evaluated for diseases like MELAS and other inflammatory conditions wherein MNRR1 levels become reduced [29]. The observation that the activation of MNRR1 can protect from inflammatory stress is crucial since patients with MELAS and other mitochondrial diseases have a higher susceptibility to infections and bacterial sepsis [30]. Hence, these results are also consistent with our recent findings that activation of MNRR1 can prevent inflammation induced preterm birth in vivo [26]. Furthermore, by identifying increased HIF2 at normoxia as the cause of reduced *MNRR1* in MELAS cells, we have uncovered a potential new therapeutic target. By contrast, most current studies target symptomatic relief such as by increasing nitric oxide levels to ameliorate stroke-like episodes [47,48].

## Data Availability

All study data presented in this manuscript are included in the article or are available from the lead contacts upon request. Unique reagents generated from this study are available from the lead contacts with a completed Materials Transfer Agreement. This study did not generate original code.

## Supplementary Materials

The following supporting information can be downloaded at: https://www.mdpi.com/article/10.3390/cells14141078/s1, Figure S1. A: Venn diagram showing activators identifed in HEK293 and MDA-MB-468 cells. B: Equal amounts of MELAS cells treated with Vehicle (DMSO) or various MNRR1 activating compounds (10 μM) for 24 h were separated on an SDS-PAGE gel and probed for MNRR1 levels. Actin was probed as a loading control and numbers below represent an average and standard deviation (SD) of two biological replicates. C-E: Equal amounts of cell lysates from various cell lines treated with vehicle (DMSO) or tizoxanide (10 μM) for 24 h were separated on an SDS-PAGE gel and probed for MNRR1 plus loading controls GAPDH or tubulin. Figure S2. A: *Above*, Chemical structures of nitazoxanide, its metabolites, and similar compounds. Abbreviations: P4, Phenyl-4-aminosalicylic acid; 4ABA, 4-Aminobenzanilide; 4ASA, 4-Amino salicylic acid; BA, Benzanilide; PBO, Phenyl benzoate; PSA, Phenyl salicylic acid; 2AN, 2-Amino 5-nitrothiazole. *Below*, Equal amounts of lysates of MELAS cells treated with Vehicle (DMSO) or the various compounds (10 μM) for 24 h were separated on an SDS-PAGE gel and probed for MNRR1 levels. Actin was probed as a loading control and numbers below represent an average and SD of 3 biological replicates. B: *MNRR1* transcript levels are shown relative to *18S rRNA* (n = 4 biological replicates, error bars represent SE). In all figures, * indicates *p* < 0.05, ** indicates *p* < 0.005. Figure S3. A: Dual luciferase reporter assay showing relative activation of *MNRR1*-luciferase levels in MELAS cybrid cells overexpressing varying proportions of empty vector (EV), and constitutively active RBPJκ (RBPJ-CA). B: Dual luciferase reporter assay showing relative activation of *MNRR1*-luciferase levels in MELAS cybrid cells overexpressing an empty vector (EV), HIF2α or constitutively active RBPJκ (RBPJ-CA) *±* HIF2α. C: Dual luciferase reporter assay showing relative activation of *COX4I2*-luciferase levels in MELAS cybrid cells overexpressing an empty vector (EV), MNRR1, and MNRR1+Auranofin (0.5 μM). Figure S4. RT-PCR for measuring *HIF2α* levels. *18S rRNA* was used as housekeeper for both analyses (n = 3 biological replicates).
