# Supplementary material for: Pseudohypoxia-Stabilized HIF2α Transcriptionally Inhibits MNRR1, a Druggable Target in MELAS
Source: Cells. 2025 Jul 15;14(14):1078. doi: 10.3390/cells14141078 (PMC12293968; doi:10.3390/cells14141078)
Supplement: Supplementary file 1 [file cells-14-01078-s001.zip › cells-3571232-supplementary.pdf]

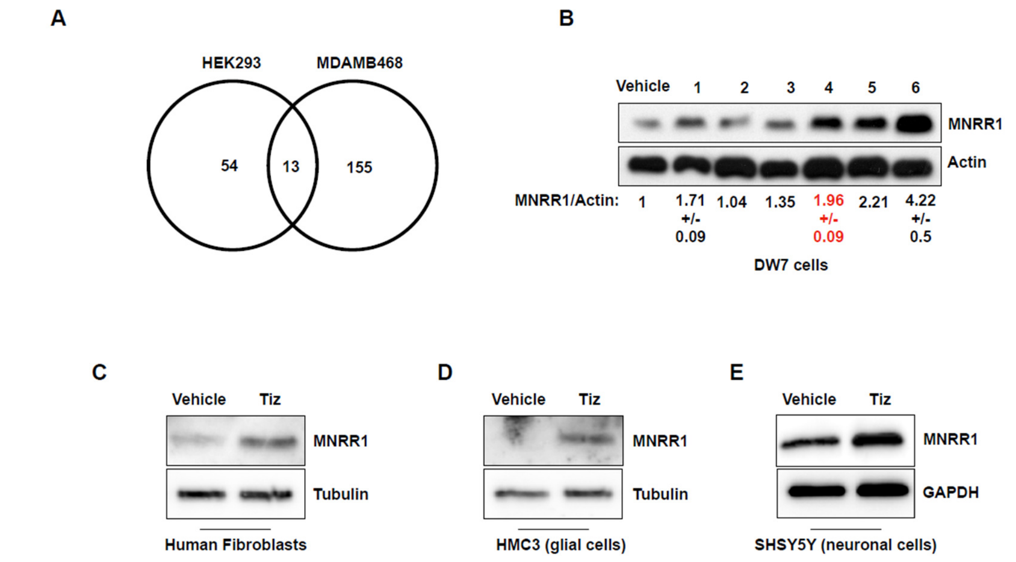

Supplementary Figure S1. A: Venn diagram showing activators identifed in HEK293 and MDA-MB-468 cells. **B:** Equal amounts of MELAS cells treated with Vehicle (DMSO) or various MNRR1 activating compounds (10 μM) for 24 h were separated on an SDS-PAGE gel and probed for MNRR1 levels. Actin was probed as a loading control and numbers below represent an average and standard deviation (SD) of two biological replicates. **C-E:** Equal amounts of cell lysates from various cell lines treated with vehicle (DMSO) or tizoxanide (10 μM) for 24 h were separated on an SDS-PAGE gel and probed for MNRR1 plus loading controls GAPDH or tubulin.

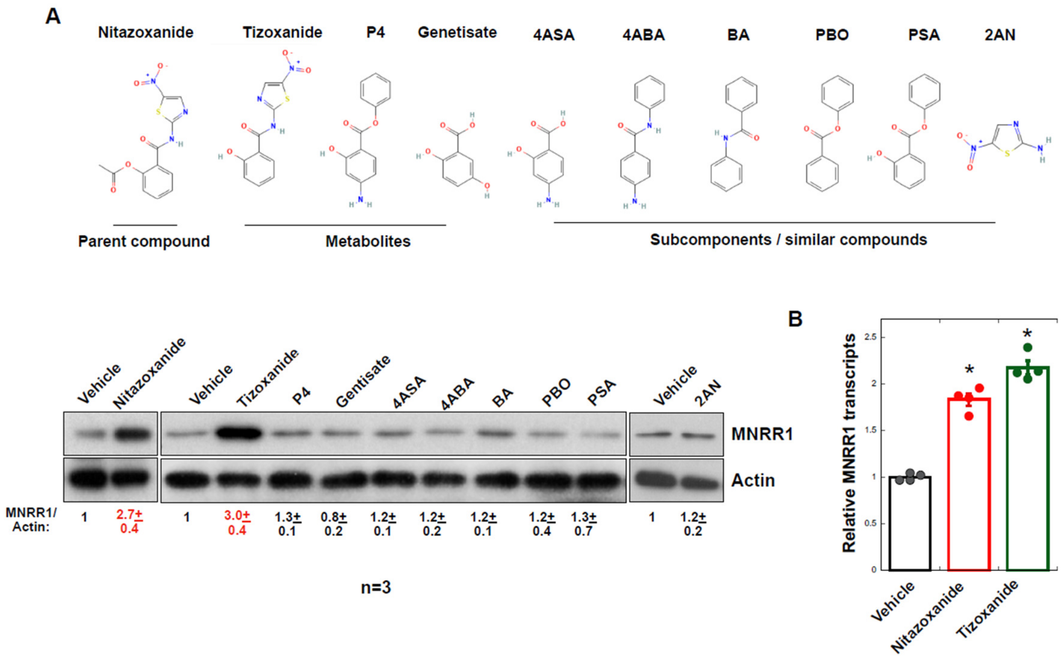

Supplementary Figure S2. A: *Above*, Chemical structures of nitazoxanide, its metabolites, and similar compounds. Abbreviations: P4, Phenyl-4-

aminosalicylic acid; 4ABA, 4-Aminobenzanilide; 4ASA, 4-Amino salicylic acid; BA, Benzanilide; PBO, Phenyl benzoate; PSA, Phenyl salicylic acid; 2AN, 2-Amino 5-nitrothiazole. *Below*, Equal amounts of lysates of MELAS cells treated with Vehicle (DMSO) or the various compounds (10 μM) for 24 h were separated on an SDS-PAGE gel and probed for MNRR1 levels. Actin was probed as a loading control and numbers below represent an average and SD of 3 biological replicates. **B:** *MNRR1* transcript levels are shown relative to 18S rRNA (n=4 biological replicates, error bars represent SE). In all figures, * indicates $p<0.05$, ** indicates $p<0.005$.

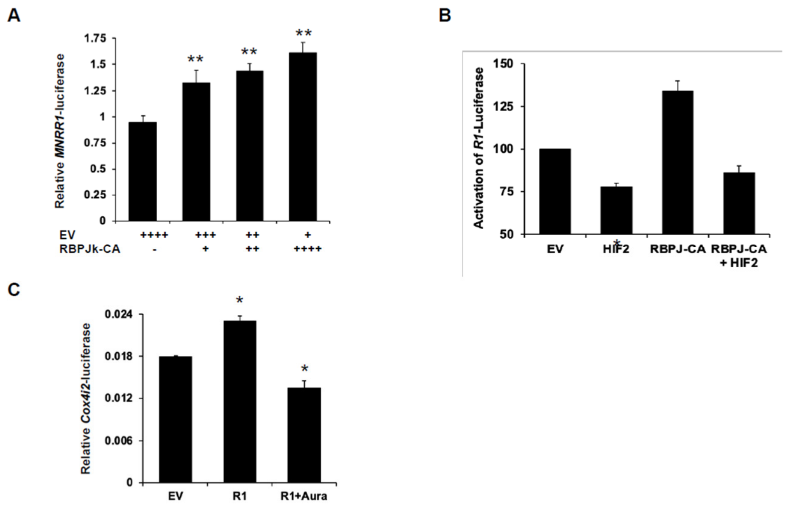

Supplementary Figure S3. A: Dual luciferase reporter assay showing relative activation of *MNRR1*-luciferase levels in MELAS cybrid cells overexpressing varying proportions of empty vector (EV) and constitutively active RBPJκ (RBP-CA). **B:** Dual luciferase reporter assay showing relative activation of *MNRR1*-luciferase levels in MELAS cybrid cells overexpressing an empty vector (EV) or constitutively active RBPJκ (RBP-CA) and HIF2α. **C:** Dual luciferase reporter assay showing relative activation of *COX4I2*-luciferase levels in MELAS cybrid cells overexpressing an empty vector (EV), MNRR1, and MNRR1+Auranofin (0.5 μM).

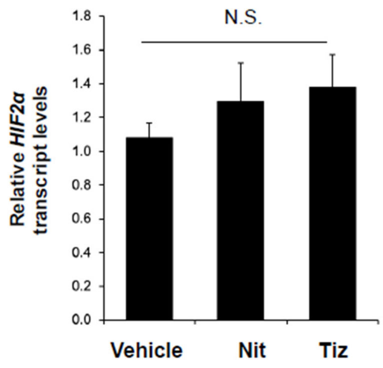

Supplementary Figure S4. RT-PCR for measuring *HIF2α* levels. 18S rRNA was used as housekeeper for both analyses (n=3 biological replicates).
